# Application of Dexmedetomidine combined with Propofol Intravenous Anesthesia in Laparoscopic Day Surgery in Pediatric Urology

**DOI:** 10.12669/pjms.38.1.4378

**Published:** 2022

**Authors:** Xiao-dan Wang, Bin Yang, Lin-lin Fan, Na Guo, Hao-bin Song

**Affiliations:** 1 Xiao-dan Wang, Department of Anesthesiology, Baoding Children’s Hospital, Baoding 071000, Hebei, China; 2 Bin Yang, Department of Surgery, Baoding Children’s Hospital, Baoding 071000, Hebei, China; 3 Lin-lin Fan, Department of Surgery, Baoding Children’s Hospital, Baoding 071000, Hebei, China; 4 Na Guo, Department of Surgery, Baoding Children’s Hospital, Baoding 071000, Hebei, China; 5 Hao-bin Song, Department of Laboratory Medicine, Baoding Children’s Hospital, Baoding 071000, Hebei, China

**Keywords:** Dexmedetomidine, Propofol, Pediatric laparoscopic surgery, Anesthetic effect

## Abstract

**Objectives:**

To evaluate the sedative and analgesic effects of dexmedetomidine combined with propofol intravenous anesthesia in laparoscopic day surgery in pediatric urology.

**Methods:**

Eighty male children with cryptorchidism and hydrocele who underwent laparoscopic daytime surgery in our hospital from January 2019 to January 2021 were selected and randomly divided into two groups: the experimental group and the control group. Children in the experimental group ranged in age from 5.7 to 11.3, with an average of 8.52±2.17 years old, while those in the control group ranged in age from 5.3 to 12.0, with an average of 8.60±2.07 years old. There were 12 cases of cryptorchidism and 28 cases of hydrocele in the experimental group, and 14 cases of cryptorchidism and 26 cases of hydrocele in the control group. Children in the control group received conventional propofol intravenous combined anesthesia, while those in the experimental group were given dexmedetomidine (2-5 ug/kg) intranasally on the basis of conventional propofol intravenous anesthesia. The anesthetic effect, analgesic effect, serum levels of inflammatory cytokines before and after surgery and adverse drug reactions in the two groups were compared and analyzed.

**Results:**

The awakening time, extubation time and retention time in the resuscitation room of the experimental group were shorter than those of the control group, with a statistically significant difference (P<0.05); The VAS pain scores of the experimental group were significantly lower than those of the control group at 15minutes, 12hour and 24hour after awakening, with a statistically significant difference (P<0.05). In addition, the levels of TNF-a, CRP, IL-6 and other inflammatory factors in the control group were significantly higher compared with those in the experimental group 24h after surgery, with a statistical significance (TNF-a, P=0.02; CRP, P=0.00; IL-6, P=0.03); The incidence of adverse drug reactions in the experimental group was 17.5%, while that in the control group was 12.5%, which was not statistically significant (P=0.53).

**Conclusion:**

Dexmedetomidine combined with intravenous propofol anesthesia may be helpful to shorten the extubation time, the recovery time and the stay time in the anesthesia resuscative room, improve the analgesic effect, and may reduce the inflammatory response and the expression of serum inflammatory cytokines, with no significant increase in side effects.

## INTRODUCTION

Cryptorchidism and hydrocele are the most common pediatric urological diseases primarily caused by congenital developmental abnormalities.^1^,^2^ Patients with this disease suffer from changes in the normal developmental environment of the testis, and abnormalities in testicular microstructures may result if left untreated, ultimately affecting spermatogenesis.^3^ Surgery is currently the main treatment for cryptorchidism and hydrocele. In the wake of the rapid development of minimally invasive technology, laparoscopy has become the standard treatment for cryptorchidism and hydrothecysis in children, featuring advantages such as less trauma, fewer concurrent symptoms and quick postoperative recovery.^4^ Day surgery, as a new surgical scheme proposed in recent years, refers to some surgeries with low risk of hospitalization on the day of surgery and discharge on the second day after surgery.^5^ Such a surgical scheme has the advantages of convenience, speed and low cost, which is very suitable for the surgical treatment of cryptorchidism and hydrocele in children. It is, however, of great significance to choose appropriate anesthetic drugs and anesthesia methods, due to the children’s younger age, poor adaptability, insufficient psychological maturity, poor treatment compliance and cooperation, and short hospitalization observation time for day surgery.^6^ Propofol is an intravenous anesthetic with the advantages of rapid onset, less irritation of the respiratory tract and rapid recovery, which is widely applied in pediatric laparoscopic surgery. However, certain adverse effects, such as restlessness during the waking period and short duration of analgesic effect, exist at the same time.^7^ Dexmedetomidine is a highly selective α2-adrenoceptor agonist, which is characterized by its obvious sedative, anti-anxiety, analgesic effects, sympathetic properties and minimal respiratory depression. It is an effective and safe drug, which is increasingly important in pediatric sedation.^8^ In this study, dexmedetomidine nasal drops combined with propofol intravenous anesthesia was applied in pediatric daytime laparoscopic surgery, obvious analgesic and sedative effects were achieved, and no adverse reactions were significantly increased.

## METHODS

### Ethical approval:

The study was approved by the Institutional Ethics Committee of Baoding Children’s Hospital on January 10, 2021(No.H-BDETKJ-SOP006-03-A/0), and written informed consent was obtained from all participants.

### Inclusion criteria:

• Children diagnosed as cryptorchidism or hydrocele via physical examination and ultrasound and other related examinations^9^,^10^;
• Children who need to be hospitalized for laparoscopic surgery;
• Children whose parents know and sign the consent form;
• Children who can cooperate and accurately describe their subjective symptoms and feelings;
• Children aged 5-12 years old.

### Exclusion criteria:

• Children with severe organic or congenital diseases of heart, liver and kidney;
• Children with allergic constitution;
• Children who have recently taken drugs that affect the study, such as hormones and immunosuppressants;
• Children who have mental or neurological disorders and cannot cooperate with the study satisfactorily;
• Children with contraindications to laparoscopic surgery.

Eighty male children with cryptorchidism and hydrocele who underwent laparoscopic daytime surgery in our hospital from January 2019 to January 2021 were selected and randomly divided into two groups: the experimental group and the control group. Children in the experimental group ranged in age from 5.7 to 11.3, with an average of 8.52±2.17 years old, while those in the control group ranged in age from 5.3 to 12.0, with an average of 8.60±2.07 years old. There were 12 cases of cryptorchidism and 28 cases of hydrocele in the experimental group, and 14 cases of cryptorchidism and 26 cases of hydrocele in the control group. The baseline data for the two groups were balanced and comparable (P>0.05). (Table-I) (It is also necessary to maintain the operative time in both groups).

**Table I:** Comparative analysis of general data between the experimental group and the control group (X̅ ±S) n=40.

Indicators	Experimental group	Control group	t/χ^2^	P
Age	8.52±2.17	8.60±2.15	0.17	0.86
Height	93.36±12.17	92.85±11.69	0.19	0.84
Weight (kg)	25.42±4.93	24.32±4.62	0.13	0.31
Number of cryptorchidism (%)	12(30%)	14(35%)	0.23	0.63

P>0.05.

### Treatment methods:

Both groups of children underwent laparoscopic surgery under general anesthesia. Laparoscopic testicular exploration and cryptorchidopexy were performed in children with cryptorchidism, while laparoscopic high ligation of processus vaginalis was performed in children with hydrocele. All patients were fasting 6h before surgery. Children in the experimental group received 0.5mL dexmedetomidine (1mg/kg) via bilateral nostrils 1h before surgery.^11^ Two groups of children were admitted to the operating room with open venous access, and then the vital signs were monitored. Tracheal intubation was performed after intravenous injection of 2mg/kg propofol, 1mg/kg midazolam, 0.2mg/kg cisatracurium and 0.2mg/kg dexamethasone. The children were inhaled oxygen of 2L/min. Propofol was injected with 5mg/(kg·h) by continuous micropump in the control group, while with 4mg/(kg·h) in the experimental group. The end-expiratory CO2 of the children was controlled to be 35-45mmHg. The oxygen flow was adjusted to 5L/min 5min before surgery, the propofol pumping was stopped after surgery, and the tracheal intubation was removed after spontaneous respiration resumed. Children were referred to the resuscitation room for observation, and returned to the general ward when the blood oxygen saturation was greater than 95% after consciousness and cessation of oxygen inhalation.

### Observation indicators:

(1) Indicators such as anesthesia recovery time, extubation time and anesthesia resuscitation room residence time were used to evaluate the anesthesia effect of the two groups of children. (2) Analgesic effect: The visual analogue scale (VAS)^12^ was used to evaluate the pain degree of the two groups 15min, 12h and 24h after surgery, and the difference in postoperative pain effect between the two groups was compared and analyzed. 0-10 points were given according to the degree of pain, the higher the score, the stronger the pain; (3) Serum inflammatory cytokines: 3mL venous blood of upper limbs was collected from all children before anesthesia and 24h after surgery. The levels of inflammatory cytokines such as tumor necrosis factor (TNF-a) and interleukin-6 (IL-6) were detected by fluorescence immunomicrospheres adsorption flow test, and immunoturbidimetry was adopted to detect C-reactive protein (CRP); (4) Incidence of adverse drug reactions: The adverse drug reactions of the two groups, including respiratory depression, bradycardia, drowsiness and restlessness, were recorded respectively.^13^

### Statistical Analysis:

All the data were statistically analyzed by SPSS 20.0 software, and the measurement data were expressed as (x̄± s). Two independent sample t-test was used for inter-group data analysis, repeated measurement analysis of variance was used for intra-group data analysis, the paired t-test was used for pairwise comparison, and χ^2^ was adopted for rate comparison. P<0.05 indicates a statistically significant difference.

## RESULTS

The comparative analysis of anesthesia effect between the experimental group and the control group is shown in Table-II, indicating that the awakening time, extubation time and retention time in the resuscitation room of the experimental group were shorter than those of the control group, with a statistically significant difference (P<0.05).

**Table II:** Comparative analysis of anesthesia effect indicators between the experimental group and the control group (X̅ ±S) n=40.

Group	Awakening time (min)*	Extubation time (min)*	Retention time in the resuscitation room (min)*
Experimental group	15.46±0.71	5.49±0.62	32.82±8.63
Control group	23.68±0.83	8.20±0.73	36.72±7.41
t	47.60	3.24	2.17
p	0.00	0.01	0.03

* P<0.05.

The VAS pain score at 24 hours after surgery was significantly lower than that at 15min after waking up, the difference was statistically significant (experimental group, P=0.02; control group, P=0.00). The scores of the experimental group were significantly lower than those of the control group at 15min after awakening, 12h and 24h after surgery, with a statistical significance (P<0.05) (Table-III).

**Table III:** Comparative analysis of analgesic effect between the experimental group and the control group (X̅ ±S) n=40.

Group	15min after awakening*	12h after surgery*	24h after surgery*	F	P
Experimental group*	4.75±0.71	3.86±0.68	2.18±0.36	17.43	0.02
Control group*	5.22±0.53	4.31±0.44	2.74±0.40	19.63	0.00
t	3.35	3.51	6.58		
p	0.01	0.00	0.00		

* p<0.05.

Prior to anesthesia, the levels of TNF-a, CRP, IL-6 and other inflammatory factors in the two groups were in the normal range, and there was no significant difference in the levels of inflammatory factors between the two groups (P>0.05). The levels of inflammatory factors in the control group were significantly higher than those in the experimental group 24h after surgery, with a statistically significant difference (TNF-a, P=0.02; CRP, P=0.00; IL-6, P=0.03) (Table-IV).

**Table IV:** Comparative analysis of changes in inflammatory factors before and after treatment between the two groups (X̅ ±S) n=40.

Group		Before anesthesia*	24h after surgeryΔ	t	P
TNF-ɑ (ng/L)	Experimental groupΔ	4.73±0.25	7.31±2.15	7.54	0.00
	Control groupΔ	4.68±0.51	9.45±3.07	9.63	0.00
	t	0.56	3.61		
	p	0.38	0.02		
CRP (mg/L)	Experimental groupΔ	5.46±3.61	9.13±2.03	5.60	0.00
	Control groupΔ	5.72±3.27	13.13±4.03	9.03	0.00
	t	0.34	5.53		
	p	0.73	0.00		
IL-6 (ng/mL)	Experimental groupΔ	116.23±43.16	134.36±41.25	2.45	0.02
	Control groupΔ	121.25±45.81	154.51±41.82	3.32	0.01
	t	0.50	2.12		
	p	0.62	0.03		

* p>0.05, Δp<0.05.

The comparative analysis of the incidence of adverse drug reactions after treatment between the two groups showed that the incidence of adverse drug reactions in the experimental group was 15%, while that in the control group was 12.5%. The incidence of adverse reactions in the experimental group was slightly higher than that in the control group, but the difference was not statistically significant (P=0.53). (Table-V)

**Table V:** Comparative analysis of adverse drug reactions between the experimental group and the control group (X̅ ±S) n=40.

Group	Respiratory depression	Bradycardia	Drowsiness	Restlessness	Incidence (%)
Experimental group	0	1	3	2	15%(6/40)
Control group	0	0	3	2	12.5%(5/40)
χ^2^					0.39
P					0.53

p>0.05.

## DISCUSSION

Cryptorchidism and hydrocele are common pediatric urological diseases with high incidence. Certain hazards will be caused to the postadolescent testicular function of patients with cryptorchidism and hydrocele if left untreated. Minimally invasive laparoscopic surgery is currently the main treatment for this type of disease, which can prevent further damage to the reproductive system of children.^13^ Day surgery is currently frequently adopted by some medical centers for this type of disease due to the short minimally invasive surgery time and low risk. Day surgery, as a new surgical concept at present, refers to a surgery with low risk of hospitalization on the day of surgery and discharge on the second day after surgery, which is characterized by simplicity, rapidity and low cost.^14^ Day surgery has stricter requirements for anesthesia, awakening, and analgesic than conventional surgery due to the short hospital stay.^16^ Consequently, reasonable selection of anesthesia methods and drugs, accurate control of dosage and depth of anesthesia are important factors to ensure the successful completion of surgery.^16^ Propofol, as a new intravenous anesthetic, boasts the advantages of no irritation, quick onset, easy to control the depth of anesthesia, and quick recovery.^17^ However, it also has some disadvantages, such as short duration, high restlessness rate in the wake period, and weak pain protection for patients.

Dexmedetomidine is a highly selective α2-receptor agonist^18^, whose pharmacological mechanism of action is to activate the α2-receptor in the presynaptic and postsynaptic central locus coeruleus to exert its hypnotic effect, thereby inducing a state of loss of consciousness similar to that of natural sleep. It is unique in that the patient remains in a state of easy awakening and cooperation. Programmed sedation of dexmedetomidine can be used as an adjuvant of anesthetics to increase the effect of anesthetics and reduce the dose of anesthetics^19^. Dexmedetomidine has a relatively long action time, which provides patients with greater comfort during and after surgery.^20^ In this study, propofol combined with dexmedetomidine was proved to have shorter awakening time, extubation time and retention time in the resuscitation room compared with conventional anesthesia scheme in pediatric laparoscopic surgery, with a statistically significant difference (P<0.05). In addition, the VAS pain score of the experimental group was significantly lower than those of the control group 15min after awakening, 12h and 24h after surgery, with a statistical significance (P<0.05).

Dexmedetomidine is the only sedative that makes patients fall asleep and wake up easily. It has the incidence of bradycardia, hypotension and respiratory depression of less than 5%, and can be corrected by reducing the dosage, infusion and other measures.^21^ Moreover, no adverse reactions are significantly increased when dexmedetomidine is used in combination with anesthetics.^22^ It was considered in the study of Shi et al.^23^ That 0.5 μg/kg dexmedetomidine can reduce the incidence of emergence delirium (ED) after sevoflurane anesthesia, and can effectively prevent the occurrence of negative post operative behavioral changes (NPOBC). It was also confirmed in our study that the incidence of adverse reactions after propofol combined with dexmedetomidine in the experimental group was 15%, while that in the control group was 12.5%. There was no increase in the incidence of adverse drug reactions compared with conventional anesthesia (P=0.53).

Dexmedetomidine, as an α 2 agonist, has a potential anti-inflammatory effect, which may not be mediated by central sedation^24^, but by a different pathway. According to animal experiments^25^, dexmedetomidine has the effect of reducing the level of pro-inflammatory cytokines in septic rats. It was considered in the study of Wang et al.^26^ That the application of dexmedetomidine during surgery reduced the secretion of cytokines during and after surgery, as well as the white blood cell count and CRP level after surgery. In a randomized clinical trial by Ohta et al.^27^, dexmedetomidine was confirmed to have a certain inhibitory effect on C-reactive protein (CRP) and procalcitonin levels in patients with sepsis. In the study by Han et al.^28^, dexmedetomidine was believed to reduce the level of postoperative inflammatory factors and improve the postoperative cognitive function and the recovery quality of anesthesia by decreasing the serum level of stress-related signaling molecules.^29^ It was also considered in the study by Liu et al.^30^ that dexmedetomidine can play a unique role not only in regulating the secretion of inflammatory cytokines, but also in effectively alleviating the pain of laparoscopic patients. According to our study, the levels of TNF-a, CRP, IL-6 and other inflammatory factors in the two groups were in the normal range before anesthesia, and increased 24h after surgery compared with those before surgery, but the increase in the control group was significantly greater than that in the experimental group, with a statistically significant difference (TNF-a, P=0.02; CRP, P=0.00; IL-6, P=0.03).

### Limitations of this study:

The sample number is small, and only the comparative analysis of indicators before and after anesthesia is involved in the study process, without strict long-term follow-up; In addition, other narcotic drugs commonly used in children have not yet been compared due to the small sample size. In view of this, proactive countermeasures are being taken to further expand the sample size, increase the follow-up content, and include other commonly used narcotic drugs in this study, so as to elaborate the effect of such a treatment regimen in more detail.

## CONCLUSIONS

Dexmedetomidine combined with intravenous propofol anesthesia may be helpful to shorten the extubation time, the recovery time and the stay time in the anesthesia resuscative room, improve the analgesic effect, and may reduce the inflammatory response and the expression of serum inflammatory cytokines, with no significant increase in side effects.

### Authors’ Contributions:

**XDW & BY:** designed this study and prepared this manuscript,and are responsible and accountable for the accuracy or integrity of the work.

**LLF & NG:** Collected and analyzed clinical data.

**HBS:** Significantly revised this manuscript.

## References

[1] Hadziselimovic F (2016). Opinion:Comment on Evaluation and Treatment of Cryptorchidism:AUA/AAP and Nordic Consensus Guidelines. Urol Int.

[2] Lundstrom KJ, Soderstrom L, Jernow H, Stattin P, Nordin P (2019). Epidemiology of hydrocele and spermatocele;incidence, treatment and complications. Scand J Urol.

[3] Docampo MJ, Hadziselimovic F (2015). Molecular Pathology of Cryptorchidism-Induced Infertility. Sex Dev.

[4] Elder JS (2016). Surgical Management of the Undescended Testis:Recent Advances and Controversies. Eur J Pediatr Surg.

[5] Bailey CR, Ahuja M, Bartholomew K, Bew S, Forbes L, Lipp A (2019). Guidelines for day-case surgery 2019:Guidelines from the Association of Anaesthetists and the British Association of Day Surgery. Anaesthesia.

[6] Shirasaka T, Kannan H, Takasaki M (2007). Activation of a G protein-coupled inwardly rectifying K+current and suppression of Ih contribute to dexmedetomidine-induced inhibition of rat hypothalamic paraventricular nucleus neurons. Anesthesiology.

[7] Dinis-Oliveira RJ (2018). Metabolic Profiles of Propofol and Fospropofol:Clinical and Forensic Interpretative Aspects. Biomed Res Int.

[8] Mohite V, Baliga S, Thosar N, Rathi N (2019). Role of dexmedetomidine in pediatric dental sedation. J Dent Anesth Pain Med.

[9] Thorup J, Cortes D (2019). Surgical Management of Undescended Testis - Timetable and Outcome:A Debate. Sex Dev.

[10] Esposito C, Escolino M, TurràF Roberti A, Cerulo M, Farina A (2016). Current concepts in the management of inguinal hernia and hydrocele in pediatric patients in laparoscopic era. Semin Pediatr Surg.

[11] Wan Z, Wang J, Cao H, Wu L (2018). Effects of different doses of dexmedetomidine on analgesic efficacy and inflammatory cytokines in patients undergoing laparoscopic surgery. Exp Ther Med.

[12] Rosas S, Paço M, Lemos C, Pinho T (2017). Comparison between the Visual Analog Scale and the Numerical Rating Scale in the perception of esthetics and pain. Int Orthod.

[13] Rahman W, D'Mello R, Dickenson AH (2008). Peripheral nerve injury-induced changes in spinal alpha(2)-adrenoceptor-mediated modulation of mechanically evoked dorsal horn neuronal responses. J Pain.

[14] de Luca U, Mangia G, Tesoro S, Martino A, Sammartino M, Calisti A (2018). Guidelines on pediatric day surgery of the Italian Societies of Pediatric Surgery (SICP) and Pediatric Anesthesiology (SARNePI). Ital J Pediatr.

[15] Strong B, Sainsbury D, Hodgkinson P, Ragbir M, Williams N (2018). Aesthetic day surgery safety in a UK facility:A 4 year retrospective study and discussion of the literature. J Plast Reconstr Aesthet Surg.

[16] Kache SA, Sale D, Ajah JL, Makama JG (2018). Paediatric day-case surgery in a new paediatric surgical unit in Northwestern Nigeria. Afr J Paediatr Surg.

[17] Filho EM, Riechelmann MB (2020). Propofol use in newborns and children:is it safe?A systematic review. J Pediatr (Rio J).

[18] Schacherer NM, Armstrong T, Perkins AM, Poirier MP, Schmidt JM (2019). Propofol Versus Dexmedetomidine for Procedural Sedation in a Pediatric Population. South Med J.

[19] Weerink MAS, Struys MMRF, Hannivoort LN, Barends CRM, Absalom AR, Colin P (2017). Clinical Pharmacokinetics and Pharmacodynamics of Dexmedetomidine. Clin Pharmacokinet.

[20] Barends CR, Absalom A, van Minnen B, Vissink A, Visser A (2017). Dexmedetomidine versus Midazolam in Procedural Sedation. A Systematic Review of Efficacy and Safety. PLoS One.

[21] Venkatraman R, Hungerford JL, Hall MW, Moore-Clingenpeel M, Tobias JD (2017). Dexmedetomidine for Sedation During Noninvasive Ventilation in Pediatric Patients. Pediatr Crit Care Med.

[22] Lin Y, Zhang R, Shen W, Chen Q, Zhu Y, Li J (2020). Dexmedetomidine versus other sedatives for non-painful pediatric examinations:A systematic review and meta-analysis of randomized controlled trials. J Clin Anesth.

[23] Shi M, Miao S, Gu T, Wang D, Zhang H, Liu J (2019). Dexmedetomidine for the prevention of emergence delirium and postoperative behavioral changes in pediatric patients with sevoflurane anesthesia:a double-blind, randomized trial. Drug Des Devel Ther.

[24] Flanders CA, Rocke AS, Edwardson SA, Baillie JK, Walsh TS (2019). The effect of dexmedetomidine and clonidine on the inflammatory response in critical illness:a systematic review of animal and human studies. Crit Care.

[25] Kang SH, Kim YS, Hong TH, Chae MS, Cho ML, Her YM (2013). Effects of dexmedetomidine on inflammatory responses in patients undergoing laparoscopic cholecystectomy. Acta Anaesthesiol Scand.

[26] Wang K, Li C (2018). Effects of dexmedetomidine on inflammatory factors, T lymphocyte subsets and expression of NF-κB in peripheral blood mononuclear cells in patients receiving radical surgery of colon carcinoma. Oncol Lett.

[27] Ohta Y, Miyamoto K, Kawazoe Y, Yamamura H, Morimoto T (2020). Effect of dexmedetomidine on inflammation in patients with sepsis requiring mechanical ventilation:a sub-analysis of a multicenter randomized clinical trial. Crit Care.

[28] Han C, Fu R, Lei W (2018). Beneficial effects of dexmedetomidine on early postoperative cognitive dysfunction in pediatric patients with tonsillectomy. Exp Ther Med.

[29] Guo Y, Sun L, Zhang J, Li Q, Jiang H, Jiang W (2015). Preventive effects of low-dose dexmedetomidine on postoperative cognitive function and recovery quality in elderly oral cancer patients. Int J Clin Exp Med.

[30] Liu X, Hu X, Li R, Zhang Y (2020). Combination of post-fascia iliaca compartment block and dexmedetomidine in pain and inflammation control after total hip arthroplasty for elder patients:a randomized control study. J Orthop Surg Res.

